# Statistical analysis of frequencies of opponents׳ eliminations in Royal Rumble wrestling matches, 1988–2018

**DOI:** 10.1016/j.dib.2018.06.023

**Published:** 2018-06-19

**Authors:** Hilary I. Okagbue, Ezinne C. Erondu, Aderemi A. Atayero, Pelumi E. Oguntunde, Abiodun A. Opanuga, Tomike I. Olawande, Ogochukwu A. Ijezie, Grace A. Eze

**Affiliations:** a Department of Mathematics, Covenant University, Canaanland, Ota, Nigeria; b Department of Electrical and Information Engineering, Covenant University, Canaanland, Ota, Nigeria; c Department of Sociology, Covenant University, Canaanland, Ota, Nigeria; d Department of Applied Data Analytics, Bournemouth University, United Kingdom; e Universita degli Studi dell׳Aquila, L׳Aquila, Italy; f Ivan Franko National University of Lviv, Lviv, Ukraine

**Keywords:** Royal Rumble, WWE, Wrestling, Smart campus, Sport analytics, Statistics

## Abstract

The datasets and their analyses presented in this paper revealed some frequencies of opponents׳ eliminations by entrance or order of elimination in Royal Rumble wrestling matches from 1988 to 2018. The frequency of eliminations by the order of entrant is quite different from order of eliminations. Statistical methods, algorithms and machine learning methods can be applied to the raw data to obtain more hidden trend not included in this article.

**Specifications Table**

**Table:** 

Subject area	Decision Sciences
More specific subject area	Sports science, Statistical data analysis
Type of data	Table and MS Excel
How data was acquired	The data was obtained from wwe.com and Wikipedia.com. About.com was helpful for the detailed dataset description.
Data format	Raw, partially analyzed
Experimental factors	Exploration of frequencies of opponents’ elimination by entrance and order of eliminations
Experimental features	20-man, 30-man and 40-man royal rumble matches were included. Greatest Royal Rumble (2018) was not included. Researchers can decide to consider only 30-man royal rumble matches.
Data source location	WWE and Wikipedia webpages [1], [2].
Data accessibility	All the data are in this data article

**Value of the data**

• The data could be helpful in prediction of winning, losing and elimination frequencies in royal rumble matches.
• The data analysis could be helpful in sports science.
• The analysis can be extended to capture the frequencies of eliminations along the brand line such as ‘ECW’, ‘RAW’, ‘SmackDowm’, ‘NXT’ and so on.
• The analysis can be done on the time spent by the wrestlers on elimination.
• Similar analysis can be done on other wrestling events such as ‘Battle royal matches’, ‘Elimination Chamber’, ‘Women׳s royal rumble’, ‘Survivor Series’ and so on.
• Advanced statistical tools can be employed to reveal hidden patterns of eliminations. Statistical hypothesis can be propounded. Such as the independence or otherwise of the entrant numbers to the order of eliminations and so on.
• Data mining tools can be used to explore the participants’ rounds of survival as a function of their entrant numbers.

## 1 Data

The data is in this article was obtained from the aforementioned websites. The data describes the frequency of eliminations by different entrants (professional wrestlers) and frequency of eliminations from the winner down to the first wrestler to be eliminated into the royal rumble matches promoted by the World Wrestling Entertainment (WWE). The data of the order of eliminations by the entrant to the last entrant is quite different from order of eliminations from the winner to the first to be eliminated.

The raw data was arranged to explore the frequencies of opponents׳ eliminations by entrance or order of elimination in Royal Rumble wrestling matches from 1988 to 2018. The data are presented as follows:

a). The frequency of eliminations by different entrants presented in Table 1 and descriptive statistics in Table 2. The raw data can be assessed as Supplementary Data 1. b). The frequency of eliminations arranged in descending order from the winner down to the first person to be eliminated. This is shown in Table 3 and the descriptive statistics in Table 4. In addition the raw data can be assessed as Supplementary Data 2.

**Table 1:** The frequency of eliminations by different entrants in Royal Rumble matches from 1988 to 2018.

Entrant	0	1	2	3	4	5	6	7	8	9	10	11	12
1	10	8	2	5		2	1	1	2				
2	16	1	7	2	2	1	2						
3	21	3	2	1	1	3							
4	20	7	1	1	1	1							
5	20	5	1	1		1	1	1			1		
6	16	10	1	2	1							1	
7	18	7	1	1		1	1	2					
8	20	5	1	3	1			1					
9	16	12		3									
10	18	8	2	3									
11	14	7	6	2		2							
12	16	9	3	3									
13	17	5	2	5	2								
14	15	11	3	2									
15	14	6	6	1	1	1	1						1
16	20	5	4	1	1								
17	16	7	5	2		1							
18	19	3	4		2		1		1	1			
19	15	4	4	2	3		2	1					
20	18	8	2	2	1								
21	18	4	7				1						
22	14	7	3	2	2	1		1					
23	16	5	5	1	2		1						
24	20	4	1		3			2					
25	20	4	2	2	1		1						
26	15	9	3	1	2								
27	18	5	3	2	1			1					
28	14	5	3	4	2	1		1					
29	17	5	3		2	1	2						
30	5	9	5	4	3	3	1						
31	1												
32	1												
33	1												
34	1												
35			1										
36		1											
37	1												
38			1										
39				1									
40		1											
Sum	501	190	94	59	34	19	15	11	3	1	1	1	1

**Table 2:** The descriptive statistics of eliminations by different entrants in Royal Rumble matches from 1988 to 2018.

Entrant	Total	Minimum	Maximum	Mean	Sum
1	31	0	8	2.129	66
2	31	0	6	1.484	46
3	31	0	5	0.935	29
4	31	0	5	0.677	21
5	31	0	10	1.226	38
6	31	0	11	1.065	33
7	31	0	7	1.194	37
8	31	0	7	0.871	27
9	31	0	3	0.677	21
10	31	0	3	0.677	21
11	31	0	5	1.129	35
12	31	0	3	0.774	24
13	31	0	4	1.032	32
14	31	0	3	0.742	23
15	31	0	12	1.548	48
16	31	0	4	0.645	20
17	31	0	5	0.903	28
18	31	0	9	1.355	42
19	31	0	7	1.581	49
20	31	0	4	1.581	22
21	30	0	6	0.800	24
22	30	0	7	1.300	39
23	30	0	6	1.067	32
24	30	0	7	1.067	32
25	30	0	6	0.800	24
26	30	0	4	0.867	26
27	30	0	7	0.933333	28
28	30	0	7	1.433333	43
29	30	0	6	1.200000	36
30	30	0	6	2.133333	64
31	1	0	0	0	0
32	1	0	0	0	0
33	1	0	0	0	0
34	1	0	0	0	0
35	1	2	2	2	2
36	1	1	1	1	1
37	1	0	0	0	0
38	1	2	2	2	2
39	1	3	3	3	3
40	1	1	1	1	1

**Table 3:** The frequency of eliminations arranged in descending order from the winner down to the first person to be eliminated in Royal Rumble matches from 1988 to 2018.

Rank	0	1	2	3	4	5	6	7	8	9	10	11	12
1st	0	2	3	6	7	2	4	3	2		2		
2nd	1	3	4	4	8	3	5	0	1			1	1
3rd	3	6	6	7	2	7							
4th	4	7	8	5	5		1	1					
5th	7	11	8	1	3			1					
6th	8	6	8	3	1	1	3	1					
7th	18	4	6	2		1							
8th	21	7	2	1									
9th	20	9	2										
10th	19	7	3		1					1			
11th	19	5	5	1		1							
12th	20	4	2	4		1							
13th	20	8	2	1									
14th	15	10	5	1									
15th	18	8	3	1	1								
16th	20	6	1	2	1			1					
17th	20	6	3	1			1						
18th	17	9	2	2	1								
19th	18	8	2	1	1	1							
20th	14	9	3	4				1					
21	13	8	3	4	1			1					
22	15	9	3	1		1		1					
23	19	7	1	2				1					
24	19	7	2	1		1							
25	21	6	3										
26	21	7		1	1								
27	23	4	1	2									
28	27	2		1									
29	25	4	1										
30	29		1										
31	1												
32	1												
33			1										
34		1											
35	1												
36	1												
37	1												
38	1												
39	1												
40	1

**Table 4:** The descriptive statistics of eliminations arranged in descending order from the winner down to the first person to be eliminated in Royal Rumble matches from 1988 to 2018.

Position	Total	Minimum	Maximum	Mean	Sum
1st	31	1	10	4.68	145
2nd	31	0	12	4.23	131
3rd	31	0	5	2.65	82
4th	31	0	7	2.29	71
5th	31	0	7	1.58	49
6th	31	0	7	2.096774	65
7th	31	0	5	0.870968	27
8th	31	0	3	0.451613	14
9th	31	0	2	0.419355	13
10th	31	0	9	0.838710	26
11th	31	0	5	0.741935	23
12th	31	0	5	0.806452	25
13th	31	0	3	0.483871	15
14th	31	0	3	0.741935	23
15th	31	0	4	0.677419	21
16th	31	0	7	0.806452	25
17th	31	0	6	0.677419	21
18th	31	0	4	0.741935	23
19th	31	0	5	0.774194	24
20th	31	0	7	1.096774	34
21st	30	0	7	1.233333	37
22nd	30	0	7	1.000000	30
23rd	30	0	7	0.733333	22
24th	30	0	5	0.633333	19
25th	30	0	2	0.400000	12
26th	30	0	4	0.466667	14
27th	30	0	3	0.400000	12
28th	30	0	3	0.166667	5
29th	30	0	2	0.200000	6
30th	30	0	2	0.066667	2
31st	1	0	0	0	0
32nd	1	0	0	0	0
33rd	1	0	2	2	2
34th	1	0	1	1	1
35th	1	0	0	0	0
36th	1	0	0	0	0
37th	1	0	0	0	0
38th	1	0	0	0	0
39th	1	0	0	0	0
40th	1	0	0	0	0

A careful examination and interpretation of Table 1, Table 2, Table 3, Table 4 could be of interest as different distinct frequencies can be obtained. For example, the top five entrant numbers with the highest number of eliminations are 1, 30, 19, 15 and 2. Moreover, charts can be drawn using the raw data.

### 1.1 Detailed data description

Royal Rumble is an annual pay per view (PPV) professional wrestling event, held every January since 1988 and organized by WWE.

The event started as a 20-man match in 1988 and 30-man thereafter, except in 2011, when it was a 40-man match. WWE network has exclusive rights for the PPV broadcast along with other current PPV events such as; *Elimination Chamber, Fastlane, WrestleMania, Backlash, Money in the Bank, Extreme Rules, SummerSlam, Hell in a Cell, TLC: Tables, Ladders and Chairs, Survivor Series, Clash of Champions.* Also other former PPV events include: *Payback, No Mercy, Battleground, Great Balls of Fire, Roadblock, Night of Champions, King of the Ring, No Way Out, Over the Limit, Vengeance, Capitol Punishment, Bragging Rights, Breaking Point, Fatal 4-Way, Judgment Day, Armageddon, One Night Stand, Cyber Sunday, Unforgiven and others.* Furthermore Royal Rumble is one of the traditional big 4 PPV events of WWE that records high event attendances and pay per views. The other three are WrestleMania, SummerSlam and Survivor Series.

Royal Rumble event often consists of Royal Rumble match, nontitle matches which can be singles, tag team, mixed tag team or special matches with stipulations and title matches. Currently the available WWE title matches are: WWE Universal Championship, WWE Championship, WWE Intercontinental Championship, WWE United States Championship, WWE Raw Women׳s Championship*,* WWE SmackDown Women׳s Championship*,* WWE Raw Tag Team Championship, WWE SmackDown Tag Team Championship and WWE Cruiserweight Championship. Royal Rumble matches are men׳s only until 2018 when the women׳s Royal Rumble was introduced.

The royal rumble match as a battle royal usually begins with entrant numbers one and two. The other competitors entered the match at a timed interval which ranges from one to two minutes until all the entrants have entered. The entrance is randomly done and the competitors assigned their corresponding entrance numbers.

The match is usually played with no definite stipulations or rules except that elimination must occur from the ring and must occur by an opponent being tossed, pushed or placed over the top rope of the ring and both feet touching the floor of the wrestling arena. That is pinfall or submission as methods of winning by the opponents does not count. The last participant that remained after all others have been eliminated is declared the winner. The only target of the participants is elimination. There is no limit on the number of eliminations. The eliminated wrestlers are prohibited from re-entering the ring. Consequently, the winners of the royal rumble match earn an opportunity for title championship of their choice at WrestleMania.

## 2 Experimental design, materials and methods

Descriptive statistics was used to sieve out some interesting frequencies of eliminations in royal rumble matches from 1988 to 2018. The data was manually retrieved from the WWE and Wikipedia websites and transferred to the Microsoft EXCEL. The data was imported to SPSS 20.0 where the various analyses were performed with the data.

All cases were included in this analysis which is 20-man, 30-man and 40-man royal rumble matches. The cases where two winners emerged were treated as one winner. The cases where the competitors were unable to compete were assigned zero elimination. However, investigators may decide to analyze the 30-man royal rumble matches.

The women׳s royal rumble matches were not considered since the event has occurred only once and hence it will take time before frequency of elimination can be studied. Also female entrant into the men royal rumbles matches were not considered.

The brand affiliation (RAW, SMACKDOWN, NXT, free agent) of the competitors were not considered since the event is independent of any brand. That is competitors from different WWE brands are drawn to compete.

Detailed statistical analysis can be performed to validate the usefulness of the data. Some examples can be seen in Refs. [3], [4], [5], [6], [7].

In Tables 1and 3, the number of eliminations by participants so far recorded in Royal Rumble matches are from 0 to 12.

From Table 1, 501 wrestlers have participated with zero elimination. Entrants numbers 3, 4, 5, 8, 16, 24 and 25 have the highest frequencies of zero elimination. It can also be seen that entrants 6, 9 and 14 have the highest frequencies of a single elimination. Again, the frequency of eliminations decreases as the number of eliminations increases from 0 to 12.

From Table 2, the top ten entrant numbers with the highest frequencies and means of eliminations are 1, 30, 19, 15, 2, 28, 18, 22, 5 and 7. Interpretation can be given to his given the entrant numbers 1 and 30. It is most likely that the first entrant often spends more time in the ring leading to more elimination while the last entrant (30-man match) comes in with vitality and eliminates other opponents whom may have been exhausted after a long time in the ring.

From Table 3, Table 4, different frequencies of elimination can be traced to the winner to the first person to be eliminated in Royal Rumble matches. From Table 4, the winner and the runners up have the highest frequency of eliminations with means of 5 and 4 respectively.

One of the significance of the data presented in this article is the comparison between winners by entrant numbers [1], [2] and the number of eliminations by entrant number. This is presented in Table 5. It can be seen that from Table 5, that generally, all the entrant numbers that have produced winners have high number of eliminations. This can be attributed as the winners being the one with the highest number of eliminations as shown in Table 4. Entrant number one (first entrant) has eliminated most opponents even though it has emerged victorious only twice. This led to the investigation of the number of eliminations for entrants that are yet to produce winners in a 30-man royal rumble match. Currently thirteen entrant numbers fall into this category and their number of eliminations are shown in Table 6. From Table 6, it can be seen that the number of eliminations are smaller when compared with Table 5 with entrants 15, 7, 6 and 11 as exceptions. This has shown that participants with high number of eliminations are more likely to be victorious when compared with those with less number of eliminations.

**Table 5:** The winners by entrant numbers and the total corresponding eliminations in Royal Rumble matches from 1988 to 2018.

Entrant number	Wins	Number of eliminations
27	4	28
30	3	64
24	3	32
19	2	49
28	2	43
22	2	39
29	2	36
2	2	46
1	2	66
23	2	32
14	1	23
38	1	2
8	1	27
5	1	38
18	1	42
3	1	29
25	1	24
13	1	32

**Table 6:** The numbers of eliminations for entrant numbers that are yet to produce winners in Royal Rumble matches from 1988 to 2018.

Entrant number	Number of eliminations
15	48
7	37
11	35
6	33
17	28
26	26
21	24
12	24
20	22
4	21
9	21
10	21
16	20
